# Minimally Invasive Endovascular Treatment With Integrated Perioperative Management in a 97-Year-Old Patient With Subarachnoid Hemorrhage: A Distal Transradial Access Case Report

**DOI:** 10.7759/cureus.108465

**Published:** 2026-05-07

**Authors:** Yu Okuma, Kentaro Shimoda, Goro Kido, Akane Tanda, Koji Yamamura, Yukihide Kagawa

**Affiliations:** 1 Department of Neurological Surgery, Sonoda Daiichi Hospital, Adachi, JPN

**Keywords:** aneurysmal subarachnoid hemorrhage, distal transradial access (dtra), frailty syndrome, japanese geriatrics, nonagenarian patient, perioperative management

## Abstract

Treatment decisions for subarachnoid hemorrhage (SAH) in nonagenarians are challenging due to the need to balance poor prognosis against treatment invasiveness. A 97-year-old woman with a premorbid modified Rankin Scale (mRS) score of 3 presented with acute-onset headache, dizziness, and vomiting. Computed tomography revealed SAH with intraventricular hemorrhage. Further imaging demonstrated a ruptured dissecting aneurysm of the left posterior inferior cerebellar artery (PICA) (World Federation of Neurosurgical Societies Grade 2, Hunt and Kosnik Grade 3, modified Fisher Group 4). Although conservative management was initially considered, endovascular coil embolization via distal transradial access (dTRA) was performed based on the family’s preference for active treatment. Targeted embolization of the dissecting “pearl” was achieved while preserving PICA flow. The procedure was completed without complications, achieving near-complete occlusion with a neck remnant. No symptomatic vasospasm or delayed cerebral ischemia (DCI) occurred. The patient was transferred back to the referring hospital with a functional status comparable to baseline (mRS score of 3). This case demonstrates that favorable early outcomes may be achieved in selected nonagenarian patients through appropriate patient selection and minimally invasive strategies. It highlights the potential value of an integrated approach combining low-profile endovascular techniques with meticulous, individualized perioperative management in the context of a super-aging society.

## Introduction

Japan is at the forefront of an unprecedented super-aging society, necessitating specialized strategies in geriatric medicine. In this context, decision-making for acute interventions in patients aged over 90 years (nonagenarians) has become a critical challenge in daily clinical practice. Subarachnoid hemorrhage (SAH) in elderly patients is generally associated with poor prognosis [1], making decisions regarding invasive treatment particularly difficult.

Clinical severity and prognosis in SAH are commonly assessed using established grading systems, such as the World Federation of Neurosurgical Societies (WFNS) scale, which is based on the level of consciousness and neurological deficits, and the modified Fisher classification, which reflects the extent of subarachnoid blood on computed tomography [2].

Furthermore, ruptured dissecting aneurysms of the posterior inferior cerebellar artery (PICA) are relatively rare and present unique therapeutic challenges, particularly when preservation of the parent artery is required. These challenges are further amplified in nonagenarian patients due to frailty, comorbidities, and limited physiological reserve.

Recent advances in endovascular techniques have expanded the availability of less invasive treatment options. In particular, distal transradial access (dTRA) has emerged as a minimally invasive approach that may facilitate early mobilization and reduce access-site complications.

Here, we report a case of a 97-year-old patient with SAH due to a ruptured PICA dissecting aneurysm who was successfully treated with coil embolization via left dTRA. This case highlights the importance of selecting an individualized treatment strategy for nonagenarian patients.

## Case presentation

A 97-year-old woman with a pre-morbid modified Rankin Scale (mRS) score of 3 [3] presented with acute-onset severe headache, dizziness, and repeated vomiting. The symptoms developed suddenly on the day of admission. On arrival, she was restless and intermittently agitated, with difficulty following commands because of pre-existing Alzheimer’s disease and acute discomfort. Nuchal rigidity was present. 

**Figure 1:**
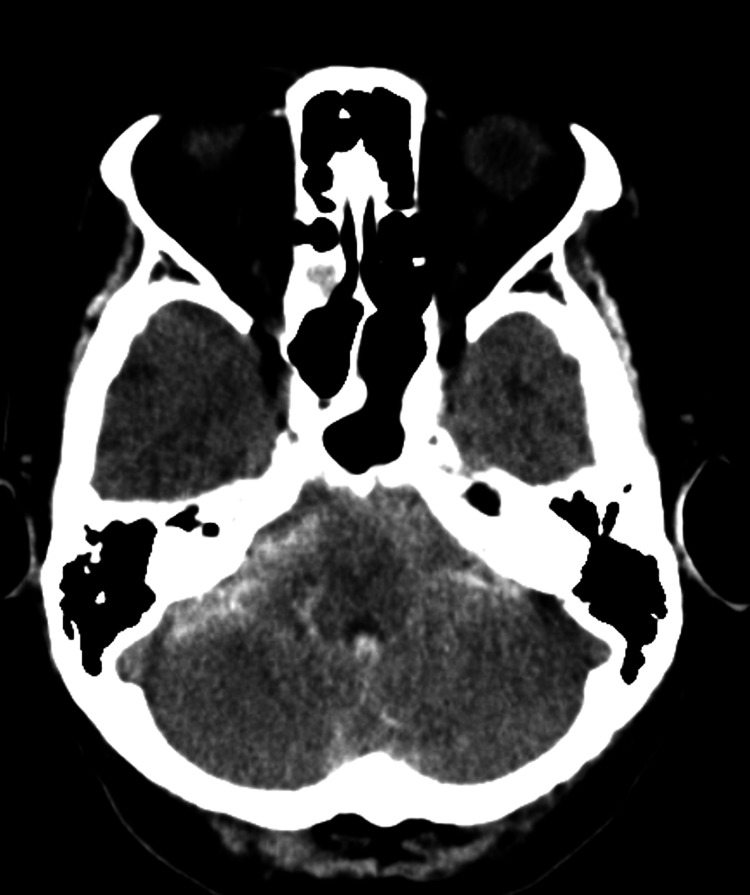
Initial axial computed tomography showing subarachnoid and intraventricular hemorrhage.

Initial computed tomography (CT) demonstrated SAH with intraventricular extension (Figure 1). Although conservative management was initially considered because of her extreme age and baseline frailty, the patient was transferred to our hospital at the request of her family for further evaluation and possible intervention.

Subsequent vascular imaging demonstrated a ruptured dissecting aneurysm of the left PICA. Clinical severity was classified as WFNS Grade 2 [4], Hunt and Kosnik Grade 3 [5], and modified Fisher Group 4 [6].

Preprocedural ultrasonographic evaluation of the upper extremity arteries demonstrated a distal radial artery diameter of 1.8 mm and a radial artery diameter of 2.8 mm on the left side, while the right distal radial artery measured 1.6 mm and the radial artery 1.9 mm. The ulnar arteries were approximately 1.0 mm bilaterally. No severe calcification or occlusive disease precluding radial access was observed. Considering vessel caliber, accessibility, and procedural ergonomics, the left side was selected for dTRA.

Her medical history included Alzheimer’s disease, diabetes mellitus treated with metformin, hypertension, dyslipidemia, and obesity (body mass index approximately 30 kg/m²).

Given the need to balance curative efficacy against procedural invasiveness, surgical trapping with bypass was considered excessively invasive in this nonagenarian patient. Therefore, a minimally invasive endovascular strategy aimed at preserving parent PICA flow was selected.

**Figure 2:**
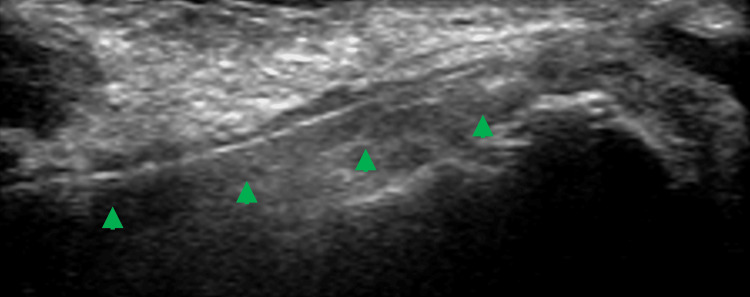
Ultrasound-guided confirmation of intravascular wire placement (green arrowhead) in the left distal radial artery using a long-axis view.

Under general anesthesia, ultrasound-guided puncture of the left distal radial artery was performed, and a 3-Fr short sheath was initially inserted. Intravascular wire placement was confirmed under ultrasound in a long-axis view (Figure 2).

**Figure 3:**
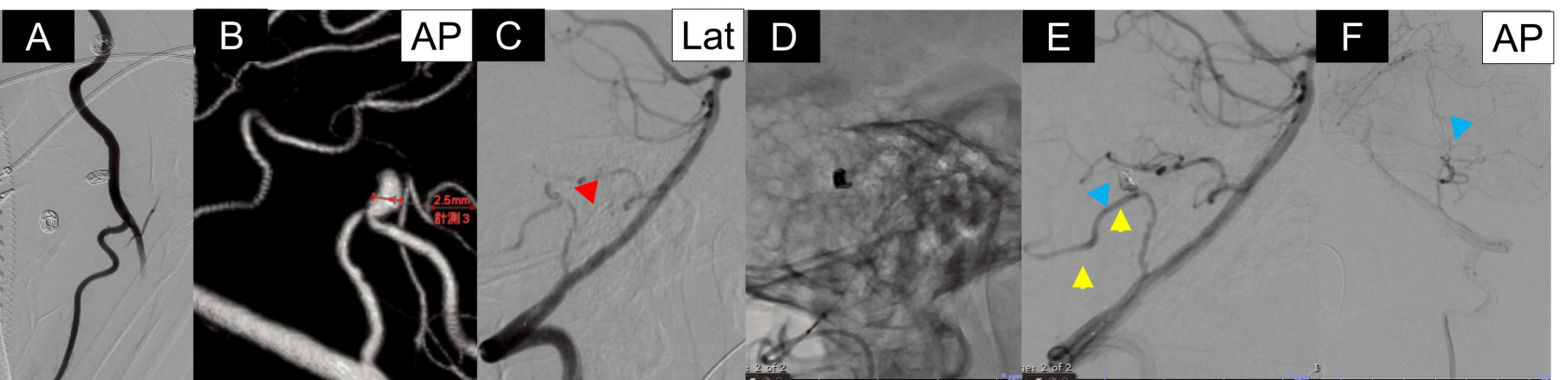
Procedural findings of distal transradial coil embolization. (A) Evaluation of radial and brachial artery anatomy during access. (B) Three-dimensional rotational angiography demonstrating a dissecting aneurysm of the left posterior inferior cerebellar artery (PICA). (C) Microcatheter positioning within the dissecting “pearl” (red arrowhead). (D-E) Coil deployment achieving near-complete occlusion while preserving PICA flow (yellow arrowhead) and improved perfusion of the involved branches (blue arrowhead). (F) Final angiography demonstrating near-complete occlusion (Raymond-Roy class II), preserved distal flow, and improved perfusion of the involved branches (blue arrowhead). Endovascular devices included a guiding catheter (Medtronic, Minneapolis, MN), a distal access catheter (Medico’s Hirata, Osaka, Japan), a microcatheter (MicroVention, Aliso Viejo, CA), a microguidewire (ASAHI INTECC, Aichi, Japan), and detachable coils (Stryker, Kalamazoo, MI). PICA, posterior inferior cerebellar artery; VA, vertebral artery; AP, anteroposterior; Lat, lateral

A vasodilator (isosorbide dinitrate) was administered intra-arterially to prevent radial artery spasm (Figure 3A). Systemic heparinization (3,000 units) was administered, resulting in an activated clotting time (ACT) of approximately 200 seconds. After initial access, the system was exchanged for a sheathless 6-Fr guiding catheter (RIST 95 cm; Medtronic, Minneapolis, MN) with a 4-Fr Simmons catheter (Medikit, Tokyo, Japan) for selective catheterization of the left vertebral artery. Navigation was achieved using a Radifocus guidewire (Terumo, Tokyo, Japan). Angiographic roadmap guidance was used to advance the system.

A distal access catheter (3.4-Fr Deflector Zero; Medico’s Hirata, Osaka, Japan) was advanced to the proximal PICA. Three-dimensional rotational angiography confirmed a dissecting aneurysm at the PICA bifurcation (Figure 3B). A microcatheter (Headway DUO; MicroVention, Aliso Viejo, CA) was navigated over a CHIKAI nexus X014 microguidewire (ASAHI INTECC, Aichi, Japan) into the dissecting *pearl* (Figure 3C). Coil embolization was performed using detachable coils (Target Tetra; Stryker, Kalamazoo, MI), with sequential framing and filling (Figures 3D-3E). Final angiography demonstrated near-complete occlusion with a neck remnant (Raymond-Roy class II), preservation of distal flow, and improved perfusion of the affected branches (Figure 3F). The volume embolization ratio was 38.5%.

**Figure 4:**
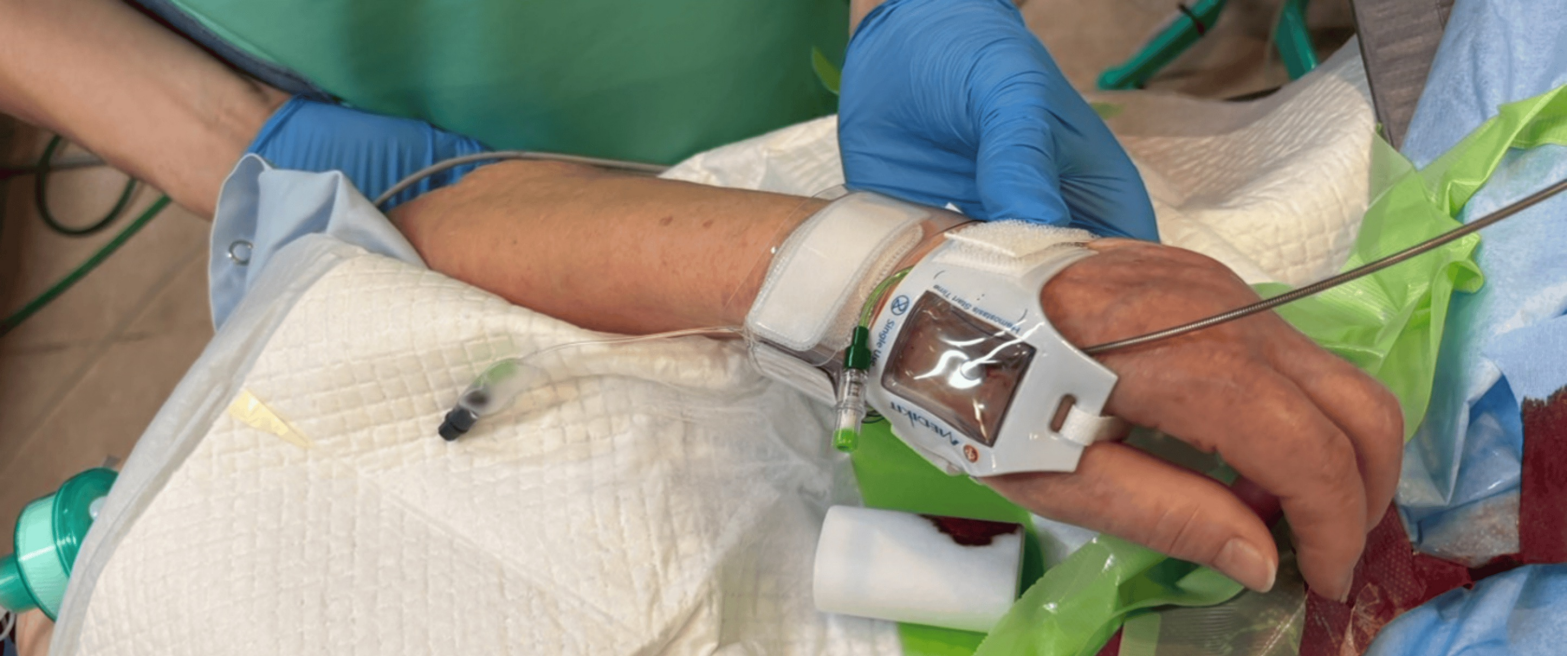
Dual radial compression devices. Hemostasis was achieved using dual radial compression devices applied at the distal puncture site and proximally over the conventional radial artery. Stepwise decompression was performed with proximal release followed by distal release under continuous pulse oximetry waveform monitoring to ensure patent hemostasis; in the event of bleeding, re-compression was applied in the same sequence with positional adjustment as needed.

Hemostasis was achieved using dual radial compression devices applied to both the distal and conventional radial artery to ensure patent hemostasis (Figure 4). In addition, a small amount of vasodilator was administered intra-arterially before catheter removal to reduce the risk of radial artery spasm and to facilitate smooth catheter withdrawal in tortuous vessels. The procedure was completed without intraoperative complications. The patient was extubated immediately after the procedure with no neurological deterioration.

**Figure 5:**
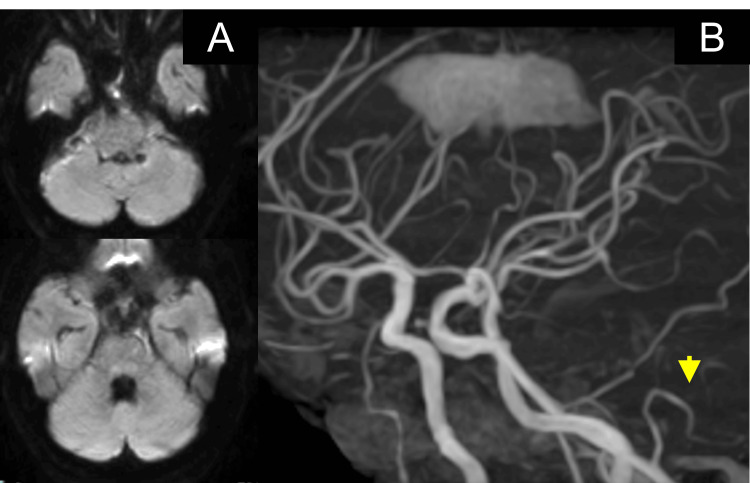
Brain magnetic resonance imaging and angiography. (A) Diffusion-weighted imaging (DWI) showing no evidence of infarction or delayed cerebral ischemia during the vasospasm phase. (B) Magnetic resonance angiography demonstrating preserved PICA flow (yellow arrowhead). PICA, posterior inferior cerebellar artery

During the vasospasm phase, magnetic resonance imaging (MRI), including diffusion-weighted imaging, demonstrated preserved PICA flow with no evidence of infarction or delayed cerebral ischemia (DCI) (Figure 5).

In addition to the procedure, multidisciplinary perioperative management was emphasized. Endothelin receptor antagonist therapy for vasospasm prevention was carefully titrated with strict monitoring of hemodynamics and fluid balance. Lipid-lowering and plaque-stabilizing therapies were administered, along with antiepileptic medications, considering the risk of seizures in elderly patients. Sedatives were carefully selected to prevent delirium.

Early rehabilitation was initiated on the same day of the procedure, facilitated by the dTRA. This strategy minimized frailty progression and preserved functional status.

The patient experienced no symptomatic vasospasm or DCI and has been transferred back to the referring hospital with an mRS score of 3, consistent with her premorbid functional status. The clinical timeline of events is presented in Table 1.

**Table 1:** Clinical timeline of events. dTRA, distal transradial access; MRI, magnetic resonance imaging; DCI, delayed cerebral ischemia; PICA, posterior inferior cerebellar artery

Day	Event
Day 0	Severe restlessness and insomnia were managed with orexin receptor antagonists, melatonin receptor agonists, and dopamine-serotonin partial agonists. A transdermal acetylcholinesterase inhibitor (donepezil patch) was also administered to support cognitive function. Suspected late-onset epilepsy was treated with brivaracetam and perampanel initiated preoperatively.
Day 1	Left dTRA coil embolization; extubation; early rehabilitation initiated
Day 2	Initiation of antiplatelet therapy and intensive lipid-lowering therapy (statins, selective peroxisome proliferator-activated receptor alpha modulators, bempedoic acid, and eicosapentaenoic acid)
Day 3	Initiation of clazosentan with careful fluid and hemodynamic monitoring; introduction of heart failure pharmacotherapy (angiotensin receptor-neprilysin inhibitors, β-blockers, mineralocorticoid receptor antagonists, sodium-glucose cotransporter-2 inhibitors, and tolvaptan)
Day 8	Follow-up angiography: no vasospasm
Day 11	MRI: no DCI; preserved PICA territory; stable clinical course

## Discussion

This case demonstrates that favorable early outcomes may be achieved in nonagenarian patients through the integration of minimally invasive endovascular techniques and meticulous perioperative management.

SAH in elderly patients is generally associated with poor outcomes [1], and conservative management is often selected. Established grading systems, including the WFNS scale and the modified Fisher classification, are widely used to assess clinical severity and predict prognosis [2]. However, recent evidence suggests that treatment decisions should not be based solely on chronological age. In patients with relatively preserved baseline functional status, active intervention may lead to improved outcomes.

Large-scale evidence, such as the International Subarachnoid Aneurysm Trial (ISAT), has demonstrated the advantages of endovascular coiling over surgical clipping in selected patients [7]. In addition, recent reports indicate that even patients over 90 years of age may benefit from endovascular treatment, emphasizing the importance of less invasive strategies [8]. In the present case, highly invasive procedures such as trapping with bypass were avoided, and targeted coil embolization was performed while preserving PICA flow, achieving a favorable angiographic result (Raymond-Roy class II) without procedural complications.

Importantly, the significance of this case extends beyond procedural success. It highlights the potential value of combining minimally invasive techniques with comprehensive perioperative management to mitigate frailty-related risks in nonagenarian patients. In this case, careful hemodynamic monitoring and individualized management allowed safe completion of treatment without vasospasm-related or hemodynamic complications.

Vasospasm management in elderly patients remains particularly challenging due to increased susceptibility to adverse effects. Poor outcomes in SAH are often associated with severe complications such as cerebral edema and secondary injury [9]. In the present case, endothelin receptor antagonist therapy was carefully titrated with strict monitoring of fluid balance and hemodynamics [10], enabling treatment without major complications. In addition, multimodal pharmacotherapy, including antiplatelet agents, lipid-lowering therapy, plaque stabilizers, antiepileptic drugs, and sedatives, was administered individually. However, this approach reflects institution-specific practice, and the evidence supporting such combinations remains limited. Potential drug-drug interactions and the difficulty in attributing clinical outcomes to specific interventions should be carefully considered.

Furthermore, the use of dTRA may offer practical advantages in elderly patients. In this case, dTRA facilitated early mobilization and same-day initiation of rehabilitation [11], which may contribute to the prevention of frailty progression. Endovascular approaches are increasingly recognized for their minimally invasive nature and favorable recovery profiles [12], making them particularly suitable for high-risk elderly populations.

The favorable early clinical course in this case likely reflects the integrated application of minimally invasive access, individualized pharmacological management, and proactive perioperative care [13]. However, the interpretation of these findings requires caution. The patient’s functional status at transfer was an mRS score of 3, comparable to the premorbid condition, but long-term outcomes, including 30- and 90-day follow-up, were not available due to early transfer. Therefore, conclusions regarding sustained functional recovery or quality of life cannot be definitively established.

This study has several limitations. First, it is a single case report, and generalizability is limited. Second, the favorable outcome may have been influenced by the patient’s relatively preserved baseline functional status. Third, follow-up duration was short, and no medium-term imaging was available to assess recurrence of the dissecting aneurysm. Fourth, the perioperative management strategy described here may not be reproducible in all institutions due to differences in expertise and available resources. Further studies are needed to validate this integrated approach in larger cohorts. These findings should be interpreted as hypothesis-generating rather than definitive evidence.

## Conclusions

Appropriate patient selection and minimally invasive strategies may lead to favorable early outcomes even in nonagenarian patients with SAH. Treatment decisions should not be based solely on chronological age but should incorporate baseline functional status and overall clinical condition. In this case, the integration of minimally invasive endovascular techniques, including left dTRA, with individualized perioperative management enabled safe treatment and early recovery without major complications. However, given the limited follow-up duration, conclusions regarding long-term functional outcomes or quality of life should be interpreted with caution. This case highlights the potential value of combining technical refinement with geriatric-focused medical management to address frailty-related risks in elderly patients, and further studies are required to evaluate the reproducibility and generalizability of this integrated approach in larger cohorts.
